# Cariogenic Microbiota and Emerging Antibacterial Materials to Combat Dental Caries: A Literature Review

**DOI:** 10.3390/pathogens14020111

**Published:** 2025-01-23

**Authors:** Jingwei Cao, Qizhao Ma, Jia Shi, Xinyue Wang, Dingwei Ye, Jingou Liang, Jing Zou

**Affiliations:** 1 State Key Laboratory of Oral Diseases, National Center for Stomatology, National Clinical Research Center for Oral Diseases, Sichuan University, Chengdu 610064, China; caojingwei1998@163.com (J.C.); mqz2080@163.com (Q.M.); shijia@stu.scu.edu.cn (J.S.); tywxyscu@163.com (X.W.); ydwkq2000@163.com (D.Y.); 2 Department of Pediatric Dentistry, West China School of Stomatology, Sichuan University, Chengdu 610041, China

**Keywords:** dental caries, microbiota, antibacterial agents, nanoparticles

## Abstract

Dental caries is the most common oral disease in the world and a chronic infectious disease. The cariogenic microbiome plays an important role in the process of caries. The ecological imbalance of microbiota leads to low pH, which causes caries. Therefore, antibacterial materials have always been a hot topic. Traditional antibacterial materials such as cationic antibacterial agents, metal ion antibacterial agents, and some natural extract antibacterial agents have good antibacterial effects. However, they can cause bacterial resistance and have poor biological safety when used for long-term purposes. Intelligent antibacterial materials, such as pH-responsive materials, nanozymes, photoresponsive materials, piezoelectric materials, and living materials are emerging antibacterial nano-strategies that can respond to the caries microenvironment or other specific stimuli to exert antibacterial effects. Compared with traditional antibacterial materials, these materials are less prone to bacterial resistanceand have good biological safety. This review summarizes the characteristics of cariogenic microbiota and some traditional or emerging antibacterial materials. These emerging antibacterial materials can accurately act on the caries microenvironment, showing intelligent antibacterial effects and providing new ideas for caries management.

## 1. Introduction

Dental caries is the most common oral disease in the world. Global disease statistics show that untreated dental caries affects 2.5 billion adults and 573 million children around the world [1], placing a heavy health burden on healthcare systems and society. Therefore, caries prevention and treatment have always been an important issue of concern. Dental caries is a multifactorial disease involving microbial, behavioral, genetic, and environmental factors [2]. As an initiating factor of caries, dental plaque plays an important role in the occurrence and development of caries.

The oral microbiota is dynamically changing because of the interactions between microorganisms, environmental exposure, and host factors. Dynamically changing bacterial communities play a very important role in maintaining human health. A variety of common human diseases are related to dysbiosis, such as inflammatory bowel disease, type 1 diabetes, etc. [3]. The balance of the microbiota in the oral cavity is also crucial to oral health. The ecological plaque hypothesis holds that during this dynamic change process, the ecological imbalance of microbial communities will lead to the occurrence of caries. The abundance of acid-producing and acid-resistant cariogenic bacteria increases. They produce organic acids such as lactic acid, formic acid, acetic acid and propionic acid during glycolysis, which lowers the pH of the local microenvironment. The low pH causes demineralization of teeth [4]. Therefore, protecting oral health microorganisms and inhibiting cariogenic microorganisms are very important for caries management.

Clinical treatment for caries including manual and mechanical rotating causes discomfort or fear for patients, especially for poorly cooperative children. Microbial factors may lead to secondary caries and failure of the treatment, so minimally invasive technologies with antibacterial effects are needed. In order to reduce the microbial factors causing caries or secondary caries, some new technologies are often used in clinical treatment to remove caries biofilms, such as the Er:YAG laser, Nd:YAG laser, etc. [5,6]. Some antimicrobial agents are also used to control the cariogenic microbiome. Broad-spectrum antibacterial materials were initially widely researched and applied, such as cationic antibacterial agents, metal ion antibacterial agents, natural antibacterial agents, etc. These antibacterial agents have good antibacterial effects, but there are also some problems. These antibacterial materials also kill microorganisms that are beneficial to health and may destroy the original microecology [7,8]. At the same time, long-term use of such agents can lead to the development of antibacterial-resistant bacteria [9]. As discoveries of materials advance, materials for the caries microenvironment or specific exogenous stimuli such as pH-responsive materials, photoresponsive materials, and piezoelectric materials are more in line with the pursuit of precision medicine and can better regulate the caries microecology. Given the complexity of microecology, researchers also focus on living materials. Probiotics from the microbiota were first selected by researchers. With the development of gene editing technology, engineered bacteria that have inhibitory effects on cariogenic bacteria have also been developed. These materials all show good, precise antibacterial capabilities and application prospects. Previous reviews mainly focused on pH-responsive materials. However, with the development of materials, there are now many emerging materials that also have good antibacterial and anti-caries effects, but there is a lack of relevant reviews.

This review summarizes the characteristics of cariogenic microbiota and the advantages and disadvantages of traditional or emerging antibacterial materials, in order to provide management strategies for inhibiting caries. The databases Medline (through PubMed) and Web of Science were searched from inception to December 2024 for the current knowledge of cariogenic microbiota and emerging antibacterial materials using the keywords “dental caries”, “microbiota”, “antibacterial agents”, and “antibacterial materials”. All articles providing information on cariogenic microbiota or antibacterial materials were selected.

## 2. Cariogenic Microbiota

Since the dysbiosis of microorganisms leads to caries, it is of vital importance to understand the characteristics of cariogenic microbiota. *Streptococcus mutans* is an early-reported and the most widely studied cariogenic microorganism. However, as the cognitive concepts of cariogenic microbiota develop, it seems that *Streptococcus mutans* is not the only cariogenic microorganism. The dynamic changes of other microorganisms other than *Streptococcus mutans* also play an important role in the process of caries. Studies have shown that highly acidic and acid-resistant species associated with dental caries include *Streptococcus mutans*, *Lactobacillus*, *Actinomyces*, *Bifidobacterium*, and *Scardovia* [10]. *Streptococcus mutans* only accounts for 0.02–0.73% of the total bacterial community [11], and approximately 15% of caries patients do not have *Streptococcus mutans* [12]. Therefore, the study of caries-inhibiting strategies should focus more on cariogenic microbiota instead of one specific microorganism.

Above all, the biodiversity of cariogenic microbiota has changed. In general, the diversity of caries microbiota is lower than those without caries [13,14]. Zhu et al. found that as dental caries progressed, the microbial diversity of children’s oral cavities decreased [15]. Microbial communities living on active caries are less abundant than those living on static caries, but they have similar beta diversity [16,17]. As caries develop, the relative abundance of caries-related species will also change [18,19,20,21,22,23]. In addition, the relative abundance of some bacteria associated with caries increases in active caries, but decreases in arrested caries [16]. An in vivo study found that in arrested caries, *Streptococcus* and *Veillonella* were more evenly distributed with other groups, while in active caries, they were dominant [24]. Overall, this change is related to a decrease in the number of aerobic or facultative anaerobic Gram-positive bacteria and an increase in Gram-negative anaerobic bacteria [25]. Changes in fungal flora are similar to changes in bacterial flora. The relative abundance of several taxa in dental caries plaque dominated by *Candida* increases significantly [26].

**Figure 1:**
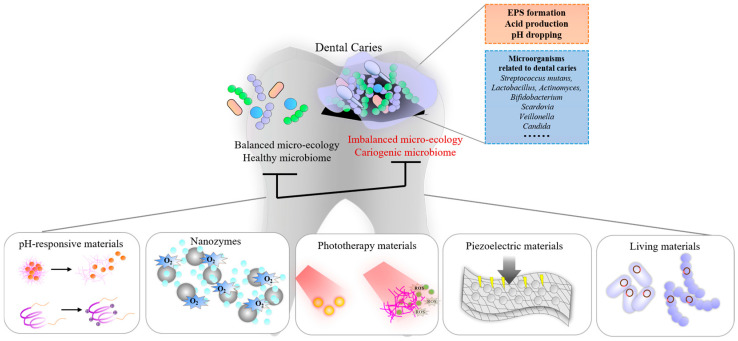
Schematic diagram of emerging antibacterial materials for dental caries.

More specifically, cariogenic microbiota differ due to individual differences, the degree of dental caries damage, etc. [10]. Microbes with high abundance in children with early childhood caries include *Prevotella amnii*, *Shuttleworthia satelles*, *Olsenella uli* and *Anaeroglobus geminatus* [27]. The microbial composition in demineralized white spots is different from that of healthy surfaces. *Actinomyces gerencseriae*, *Actinomyces naeslundii*, *Actinomyces israelii*, *Actinomyces viscosus*, *Prevotella nigrescens*, *Dialister micraerophilus*, *Eubacterium_XI G 1 infirmum*, *Streptococcus sp_Oral_Taxon_065*, and *Corynebacterium matruchotii* are more abundant on the surface of the initial enamel lesion [28]. There are also differences in the microorganisms between enamel caries and dentin caries. *Lactobacillus* species are only detected in dentin caries, while a higher abundance of *Prevotella* is found deep in dentin [29]. Richards et al. believed that *Streptococcus mutans*, *Scardovia wiggsiae*, *Paradovia denticolens* and *Lactobacillus salivarius* only exist in dentin caries [30] (Figure 1).

Based on the complexity of microbial communities, interactions between microorganisms may aggravate or inhibit the occurrence of caries, so it is also important to study the interactions between microorganisms. Different microorganisms may interact through synergy or antagonism. An example of synergy in the oral cavity is the collective degradation of salivary glycoproteins by microorganisms. The complementary enzyme activity allows the use of mucin in saliva as an energy source, because no microorganism possesses the diverse array of enzymes needed for their complete breakdown [31]. In addition, in the food chain, examples of metabolites from one species being used as primary energy by a partner species have also been documented [32]. Antagonistic effects mediated by the production of bacteriocins and hydrogen peroxide may also affect community aggregation [33]. At present, the most studied interaction between microorganisms is the relationship between *Streptococcus mutans* and other cariogenic microorganisms. Proteus secretes an external enzyme called glucosyltransferase (Gtfs), which converts sucrose into extracellular glucan, which is the main component of EPS. This extracellular glucan enhances the adhesion of bacteria to the tooth surface and facilitates the copolymerization of bacteria with other bacteria to form a cariogenic biofilm [34]. A new cross-feeding mechanism mediated by GtfB was found in mixed biofilms of *Streptococcus mutans* and *Candida albicans* that can enhance the sugar metabolism level in the mixed biofilms and enhance the cariogenic toxicity of the biofilms [35,36]. Based on the understanding of the dental caries microbiome, dental caries is considered to be a disease related to an imbalance in dental caries microecology. Therefore, more and more research has focused on microecological regulation or precise antibacterial research, rather than killing all bacteria.

## 3. Traditional Antimicrobial Materials

Dental plaque is the initiating factor of caries. Inhibiting the growth of cariogenic microorganisms and inhibiting plaque can reduce the occurrence of caries. The use of antibacterial agents is an important way to inhibit microorganisms. Studies have proven that they have good antibacterial and anti-caries effects. Materials mainly include cationic antibacterial agents, metal ion antibacterial agents, natural antibacterial agents, etc.

Cationic antibacterial agents bind to negatively charged bacterial cell membranes through positive charges to disrupt the electrical balance, and some cationic antibacterial agents can also promote bacterial lysis by binding to bacterial cell membranes [37,38]. Chlorhexidine is one of the earliest cationic antibacterial agents used to treat dental caries, and is still the gold standard for many anti-plaque experiments [39]. Chlorhexidine exerts antibacterial effects by destroying cell membranes. Experiments have shown that chlorhexidine has antibacterial effects on both Gram-positive and -negative bacteria, facultative anaerobic bacteria, and aerobic bacteria [40,41]. It also reduces dental plaque adhesion by acting on the acidic groups of glycoproteins in saliva and reducing the binding of bacteria to the tooth surface [42]. Quaternary ammonium is another common cationic antibacterial agent. Short-chain quaternary ammonium monomers exert antibacterial effects through positively charged ammonium groups. Long-chain quaternary ammonium compounds have dual killing properties. Due to the increase in alkyl chain length, the hydrophobicity of quaternary ammonium compounds increases, thereby improving their ability to penetrate hydrophobic bacterial cell membranes [43,44]. Quaternary ammonium has significant inhibitory effects on cariogenic bacteria such as *Streptococcus mutans*, *Actinomyces* and *Candida albicans* [45,46], and disinfectants containing 5% quaternary ammonium are more effective than chlorhexidine in eradicating dentin bacteria [47,48].

The research on metal ion antibacterial agents is also quite extensive, including Ag^+^, Cu^2+^, Zn^2+^, etc. Metal ion antibacterial agents bind to the anionic components of microbial cell membranes, resulting in leakage of cell contents, loss of cell mobility, and cell death [49]. Ag^+^ destroys metabolic enzymes and blocks the electron transport system, inactivating bacterial DNA and RNA [49]. Recently, the application of metal ion nanoparticles has become a hot research trend. Silver nanoparticles (AgNPs) can significantly inhibit the growth of *Streptococcus mutans* [50]. Ag^+^ has already been widely used clinically. Silver diamine fluoride (SDF) is used to prevent the progression of dental caries in children [51,52]. ZnO nanoparticles inhibit the growth of *Streptococcus mutans*, and their antibacterial properties are mainly due to reactive oxygen species (ROS). The release of ROS causes oxidative stress on the bacterial cell wall, ultimately leading to its lysis [53]. CuO nanoparticles can inhibit the growth of *Candida albicans* and *Streptococcus oralis*. Imani et al. synthesized HA–CuO–TiO_2_ nanocomposites and found that TiO_2_, CuO, and HA nanoparticles had the greatest contribution to reducing bacterial survival [54,55].

Some natural antibacterial agents extracted from various natural products, such as curcumin, honey, xanthazole, green tea extracts, aloe vera, etc., have the advantages of low toxicity, wide availability, and low cost. Honey exerts antibacterial effects through low pH, high osmotic pressure, hydrogen peroxide, gluconic acid and antibacterial peptides [56,57,58]. It inhibits both Gram-positive and Gram-negative bacteria [59,60]. Licorice extract has a higher inhibitory effect on oral pathogens than sodium fluoride [61]. Adding plant extracts to oral care products such as toothpaste, mouthwash, and oral care functional foods can enhance their anti-caries properties [62]. Xanthazole can form hydrogen bonds between hydroxyl groups and proteins in the cell membrane and interact with the cell membrane of *Candida albicans*. It will affect membrane permeability and ultimately lead to fungal lysis [62]. Therefore, natural plant extracts can be used as an adjunct therapy for oral biofilm management.

The traditional antibacterial materials have broad-spectrum antibacterial effects and inhibit the growth of caries biofilms, but they also have inhibitory effects on microorganisms that are beneficial to increasing the pH of the local microenvironment. Furthermore, studies have reported that some bacteria are resistant to cationic antibacterial agents such as chlorhexidine and quaternary ammonium compounds [40,63]. The bacterial resistance increases the difficulty of follow-up treatment. Metal ion antibacterial agents may discolor teeth, which affects aesthetics. Some in vivo studies have shown that metal ions show influence on thrombosis, myocardial infarction, and inflammation [64]. The biological safety of long-term use is still questionable. Because of the disadvantages of traditional antibacterial materials, it is necessary to develop new materials that are biologically safe and exert precise effects on cariogenic biofilm.

## 4. Intelligent Antibacterial Materials

Recently, intelligent antibacterial materials precisely affecting the caries microenvironment have become a hot topic. These materials change responsively according to the caries microenvironment or specific triggering systems. These changes make materials with no antibacterial effect antibacterial. Unlike traditional antibacterial materials, intelligent antibacterial materials only exert antibacterial effects under certain circumstances. A number of studies have shown that this targeted antibacterial ability gives new materials certain potential for bacterial community regulation and can effectively reduce the cariogenic ability of oral microorganisms (Table 1).

### 4.1. pH-Responsive Materials

In the caries microenvironment, the local acidic environment caused by an imbalance of flora is the main cause of demineralization of enamel. Therefore, based on the characteristics of this process, researchers designed materials based on the characteristics of low pH. The materials are activated under acidic conditions and achieve the purpose of precise antibacterial effects.

pH-responsive materials can exert antibacterial effects under acidic conditions, but their antibacterial properties disappear in neutral environments. These materials mainly comprise pH-responsive antibacterial agents and pH-responsive drug delivery systems. It has been found that pH-responsive antibacterial agents that can be used to regulate caries biofilms mainly include antimicrobial peptides (AMPs), tertiary amines, iron oxide nanoparticles, etc. [97].

AMPs are endogenous biological molecules that can be extracted or synthesized from natural compounds. They have a wide range of antibacterial activity and can kill viruses, fungi, and Gram-negative and Gram-positive bacteria. Some of them are pH-responsive and are called pH-activating peptides. Zhang et al. found that pHly-1 nanoparticles bind to bacterial membranes and undergo a helix–helix conformation transition under acidic conditions, thereby penetrating the lipid bilayer and destroying the biomembrane. However, at neutral pH, beta sheet pHly-1 nanofibers remain aggregated and have a negligible inhibitory effect on biofilm activity. Treatment with pHly-1NPs inhibited saliva biofilm growth in vitro and caries in rats in vivo. Analysis of the oral microbial community of rats found that pHly-1NPs can effectively inhibit oral diseases without affecting the composition of the oral microbiota [65]. The mechanism may be related to the increased expression of genes that regulate cell lysis by *Streptococcus mutans* and the killing effect of cell membranes. Quorum sensing-related genes, which are related to bacterial proliferation and regulation of herd behavior, have been downregulated [98]. GH12 and LH12 are other pH-activating peptides that inhibit exopolysaccharide synthesis, water-insoluble glucan synthesis, and lactic acid production in *Streptococcus mutans* biofilms under acidic conditions. The proportion of *Streptococcus gordonis* increased in the dual-strain biofilm model, while the proportion of *Streptococcus mutans* decreased, demonstrating the potential of pH-activating peptides for flora regulation [66,67].

The tertiary amine (TA) materials dodecylmethylaminoethyl methacrylate (DMAEM) and hexadecylmethylaminoethyl methacrylate (HMAEM) can transfer to quaternary ammonium because of protonation under acidic conditions, and then play a contact killing effect. These materials undergo deprotonation under neutral conditions and do not exert antibacterial effects. TA-modified adhesive or resin has a good effect on inhibiting caries, and can also increase the diversity of saliva biofilms, which is conducive to the health of the oral flora [68,69]. Yang et al. treated rats with caries with DMAEM monomers. The results showed that oral microbial diversity increased and the relative abundance of oral probiotics such as *lactobacillus* increased, indicating that DMAEM has the potential to maintain the balance of oral flora [99]. These pH-responsive antibacterial materials kill bacteria at low pH, which reduces the acid production of cariogenic microbiome. When the pH increases, the killing effect disappears, which is conducive to the aggregation and growth of a healthy microbiome.

pH-responsive drug delivery systems are another class of materials that can effectively exert antibacterial effects in the acidic microenvironment of caries. The carrier usually contains specific functional groups that can respond to the changing pH value of the surrounding environment. The reaction mechanism of the carrier to pH mainly includes charge transfer of pH-responsive residues and degradation of degradable residues under acidic conditions [100]. pH-responsive nanocarriers are the most widely used drug delivery system in the oral cavity [101], including dimethylaminoethyl methacrylate (DMAEMA), polyethylene glycol (PEG), chitosan, mesoporous silica nanoparticles (MSNs), etc. [102]. Horev et al. found that due to the protonation of the amino group of DMAEMA under acidic conditions, the hydrophilic nature of DMAEMA enhances the inhibitory effect of the hydrophobic antibacterial drug it carries on biofilms [70,71]. The study found that DMAEMA particles loaded with farnesol reduced the viability of *Streptococcus mutans* biofilms by 80%, while the viability of the free farnesol-treated group was only reduced by 20% [70,71]. Peng et al. found that there was no significant difference in the antibacterial properties of DMAEMA-loaded chlorhexidine and free chlorhexidine, both of which reduced the activity of biofilms and lactic acid production. However, compared with free chlorhexidine, DMAEMA-loaded chlorhexidine had the same anti-caries effect, reducing the damage of chlorhexidine to the microecology [72]. The abundance of *Peptostreptococcus* in the normal oral flora is higher, and it is speculated that DMAEMA loading chlorhexidine is more conducive to microecological balance and health [72]. PEG-loaded chlorhexidine, bedaquiline, sodium fluoride, farnesal, etc. all showed inhibitory effects on *Streptococcus mutans* [73,74,75,76]. Xu et al. coupled PEG with salivary protein peptides to promote nanocarriers to directly adhere to the surface of the tooth enamel, maximizing the effect of the drug on dental plaque, reducing the proportion of *Streptococci* in the oral cavity of animal models, and exerting a bacterial community regulation effect [75]. Chitosan protects histones from being decomposed in saliva and release histones in the acidic microenvironment of caries, effectively inhibiting the growth of *Streptococcus mutans* [77]. MSNs are inorganic porous materials that are endowed with pH responsiveness by surface modification [103,104]. Studies have found that poly(L-glycolic acid) (PGA)-modified MSNs equipped with chlorhexidine have inhibitory effects on *Streptococcus mutans* and *Staphylococcus aureus* in an acidic environment [78], and CHX-loaded, silver-decorated MSNs can inhibit the growth of *Streptococcus mutans* in an acidic environment [79]. These antibacterial agents show antibacterial effects at low pH. However, their antibacterial effects disappear at neutral pH, which gives non-acid-resistant bacteria a chance to grow. They are less toxic to a normal oral microbiome than traditional agents, so they may help the microbiome transfer from a cariogenic state to a healthy state. Furthermore, using nanoparticles to encapsulate a drug is also a way to accurately act on the cariogenic microbiome. After being loaded on nanocarriers, such materials have the advantages of precise release and local bacterial regulation and have great application prospects.

### 4.2. Nanozymes

Nanozymes are a class of nanomaterials that exhibit simulated enzyme activity, and their catalytic properties can selectively treat various clinical symptoms associated with chronic oral infections [105]. Nanozyme therapies with peroxidase activity have been the most widely used in the treatment of caries. This kind of therapy promotes the decomposition of hydrogen peroxide to produce ROS with antibacterial activity, which is highly destructive to cell surfaces, biomembrane substrates, and microbial cells [80,106]. Iron oxide is a common catalase, so researchers have developed various iron oxide nanoparticles. With the deepening of research, it was found that this material can not only inhibit cariogenic bacteria but also degrade the matrix to inhibit cariogenic biomembrane. Catalytic nanoparticles (CAT-NPs) containing biocompatible Fe_3_O_4_ can effectively restrict the growth of *Streptococcus mutans* and exploit GtfB and GtfD in the matrix, effectively reducing the occurrence of dental caries in rats [80]. Huang et al. found that ferumoxytol nanoparticles effectively inhibited the growth of *Streptococcus mutans* in in vitro experiments of mixed-species biofilm models, but had negligible impact on *Streptococcus oralis*. They had no adverse impact on the diversity of oral microbial communities and showed good bacterial regulation ability [81,107].

Nanoparticles were modified according to the characteristics of caries biomembrane to enhance specificity in order to better achieve precise treatment effects. Naha et al. developed dextran-coated iron oxide nanoparticles termed nanozymes (Dex-NZM) to target biofilms with high specificity. The dextran coating promoted the incorporation of NZM into the exopolysaccharide structure and binding within the biofilms, thereby activating H_2_O_2_ for local bacterial sterilization and exopolysaccharide matrix decomposition. There were no changes in the microbial composition or diversity in the rat oral cavity after treatment, indicating that the material can prevent dental caries without affecting the microbial diversity in the rat oral cavity [82].

Recently, multifunctional nanozymes have also become a research hotspot. Huang et al. combined ferumoxytol nanoparticles with stannous fluoride (SnF_2_), which significantly inhibited the formation of biomembrane and promoted the remineralization of tooth enamel [83]. Zhang et al. built a DNA-encoded nanozyme sensor array and diameter-dependent nanozyme sensor array to identify bacteria, including *Streptococcus mutans*, *Streptococcus salivarius*, *Lactobacillus acidophilus*, etc. This material not only accurately identified bacteria but also effectively eliminated cariogenic bacteria [84]. Silver particles were anchored onto nanoparticles composed of L-cysteine and graphdiyne (GDY/L-cys/Ag (GLA) nanozymes). GLA exhibits peroxidase-like activity activated by acidic plaque biofilms, while simultaneously enhancing Ag release triggered by acidic plaque biofilms, producing additional reactive oxygen species. The material can inhibit the growth of *Streptococcus mutans* biofilms and the production of EPS. At the same time, the material has been confirmed to have a remineralization function and is a multifunctional intelligent material [85].

Nanozymes are often used in conjunction with hydrogen peroxide or with bacteria that produce hydrogen peroxide. Iron oxide nanozymes or iron sulfide nanozymes were used in combination with *Streptococcus gordonii*, and effectively disrupted the formation of *Streptococcus mutans* biofilms [108]. Nanozymes have good antibacterial effects and the advantage of accurately acting on target sites. At the same time, by editing nanozymes, multiple functions can be achieved. Nanozymes with bacterial recognition function help integrate caries diagnosis and treatment.

### 4.3. Photoresponsive Materials

Antibacterial photothermal therapy (aPTT) based on near-infrared (NIR) light-triggered wavelengths between 700 and 1400 nm is a new antibacterial strategy. aPTT is a non-invasive local treatment that relies on harmless light to activate non-toxic or low-toxicity photosensitizers (PSs), which convert light energy into heat. Hyperthermia may evaporate cytosol, lyse microbial cell membranes, and trigger protein denaturation, leading to microbial cell death. It has the advantages of deep tissue penetration and no drug resistance [109]. Xu et al. loaded the surface of Fe_3_O_4_ nanoparticles with Ag by polydopamine (PDA) reduction and then grafted glycol chitosan (GCS) after a second PDA coating. FePAgPG released Ag under NIR light to accurately achieve antibacterial functions. At the same time, the material slowed the release of Ag through PDA, effectively reducing the damage caused by the excessive release of Ag to tissues [86]. Black phosphorus nanoparticles can mediate photothermal antibacterial activity and promote remineralization, but cannot adhere to tooth surfaces for a long time. Ran et al. constructed a multifunctional hydrogel dressing (BP@CP5) through the physical loading of BPNs within catechol-modified chitosan (CHI-CS) and PLGA-PEG-PLGA (PPP) hydrogels. BP@CP5 showed good wet adhesion and the ability to inhibit *Streptococcus mutans* and *Streptococcus sanguis* biofilms, and promoted remineralization by degrading artificial saliva to produce phosphate ions [87]. aPTT has limitations in precise temperature control and increases the risk of thermal damage to adjacent healthy tissues or organs connected to the biofilm. Combining a temperature-sensing system with aPTT will help reduce the side effects of PTT.

Antibacterial photodynamic therapy (aPDT) is another phototherapy method that produces cytotoxic substances through PSs to clear pathogens. This process produces ROS, leading to damage to bacterial membranes and cell walls, destruction of lipids, proteins and ion channels, removal of key metabolic enzymes, cell aggregation, and direct inhibition of exogenous virulence factors such as lipopolysaccharides, collagenases and proteases [110]. For instance, methylene blue (MB)-mediated aPDT can effectively inhibit *Streptococcus mutans*. At the same time, structural and storage polysaccharides from *Streptococcus mutans* mature biofilms were impaired by aPDT [111]. Many PSs currently developed are based on PSs approved by the U.S. Food and Drug Administration, and have greater potential for clinical applications. However, some PSs have hydrophobic characteristics, which is not conducive for them to penetrate or stay in cariogenic biofilms. Therefore, PSs carried by nanomaterials have become a research direction to enhance the effect of aPDT. Liu et al. developed a bioresponsive nanoparticle loaded with chlorine e6 (MPP-Ce6). MPP-Ce6 enhanced the hydrophilicity of the material and its ability to penetrate biomembrane. In an acidic environment, the nanoparticles released the carrier Ce6, and the growth of *Streptococcus mutans*, *Streptococcus sobrinus* and *Streptococcus sanguis* biofilms was inhibited after 660 nm illumination [88]. Both chitosan nanoparticles (CNPs) and titanium dioxide nanoparticles (TiO_2_NPs) carrying MB can enhance the efficacy of aPDT, and their modified adhesives both inhibit the growth of *Streptococcus mutans*. However, MB-CNPs show better antibacterial effect than MB-TiO_2_NPs, which may be related to the lack of the chelation properties of TiO_2_NPs [89]. Nanoparticles and nanoparticle-loaded membranes based on chitosan/sodium alginate and curcumin (CUR) have also shown potential to inhibit the growth of *Streptococcus mutans*, but have insufficient antibacterial effect on *Candida albicans* [90]. Therefore, the dose of PS applied to aPDT needs to be further explored. Current research is mostly limited to a single-strain biological model of *Streptococcus mutans*. In-depth research needs to be conducted due to the complexity of caries microbiota.

Some photosensitive materials also exhibit aPDT/aPTT functions and strong biomembrane dispersion capabilities. A pH-responsive polyethylene glycol (PEG)-coated IR780 nanomaterial and CIP were released in an acidic environment and activated when exposed to 808 nm near-infrared light, producing local hyperthermia and cytotoxic ROS, cooperating with CIP to eradicate *Streptococcus mutans* biofilms, and effectively inhibiting caries in rat models [91]. Zhang et al. designed an adaptive supramolecular nanoformulation composed of guanidinium-modified calix arene grafted with fluorocarbon chains (GC5AF5) and zinc phthalocyanine tetrasulfonate (ZnPcS4). Its photothermal antibacterial properties led to the rupture of bacterial cell membranes and the release of intracellular ATP. Subsequently, the competitive inclusion of ATP triggered the liberation of ZnPcS4, which converted aPTT into aPDT, produced cytotoxic singlet oxygen, and accelerated the clearance of oral bacterial biofilms [92]. A variety of semiconductor materials also exhibit aPTT/aPDT properties. A 5% wt nanostructured graphene oxide (nGO)-modified orthodontic composite inhibited the growth of *Streptococcus mutans* biofilms, *gtfB* expression, and the metabolic activity of *Streptococcus mutans*. Moreover, the material still showed inhibitory effects on *Streptococcus mutans* after 150 days of artificial saliva washing, indicating that it effectively made up for the shortcoming of insufficient long-term performance of PSs [112]. MXene is also a potential PS for aPTT/aPDT due to its high photothermal conversion efficiency and high absorption in the near-infrared region. Studies have shown that MXene-mediated aPTT/PDT has strong inhibitory effects on *Staphylococcus aureus*, *Escherichia coli* and some other bacteria [113]. MXene has been widely studied in the medical field, but research on its application in caries microbiota is still lacking. In addition to exerting antibacterial effects through aPTT/aPDT, these semiconductor materials also have piezoelectric properties and can exert antibacterial effects under different stimuli.

### 4.4. Piezoelectric Materials

Piezoelectric materials generate charges in response to applied mechanical loads, and antibacterial through electrical stimulation is another type of bacterial population regulation method with strong controllability. Electrical stimulation can be based on the direct contact theory. It directly causes bacterial death by destroying the integrity of the cell membrane [114]. The theory of indirect killing is that electrical stimulation produces a toxic substance or triggers changes in pH and temperature and current fluctuations [114]. It is not enough to rely solely on the piezoelectric effect of piezoelectric crystals to generate ROS to eradicate bacteria. Therefore, the polarization of piezoelectric materials can be enhanced by building material surfaces and interfaces to promote carrier migration [115]. The improved piezoelectric material shows extremely strong antibacterial effect. Zinc oxide nanorod@graphdiyne nanosheets (ZnO@GDY NR) with unparallel piezocatalytic enzyme mimic activity have peroxidase-like activity and high-pressure electrical response, showing almost 100% antibacterial efficacy against multidrug-resistant pathogens of methicillin-resistant *Staphylococcus aureus* and *Pseudomonas aeruginosa* [116]. The polycrystalline nanomaterial barium titanate (BaTiO_3_) modified with AuNPs has an antibacterial rate of 99.2% against *Staphylococcus aureus* [117]. Two-dimensional semiconductor materials such as GO and MXene have layered structures with surface charges, allowing them to be connected to piezoelectric nanomaterials through electrostatic interactions, facilitating the separation and transfer of charge carriers. A ZnO–GO nanocomplex has excellent antibacterial activity against *Escherichia coli* and can also degrade organic dye pollutants in the dark using ultrasound-driven piezoelectric catalysis [118]. BiFeO_3_/MXene improves the utilization efficiency of polarization and photogenerated charges generated by MXene, thereby increasing the yield of ROS under reaction conditions. It can be used for rapid and effective treatment of osteomyelitis [119]. A biomass aerogel composite containing BaTiO_3_ nanoparticles and MXene shows good piezoelectric sensing properties and photothermal antibacterial effect, and has complete bactericidal effect on *Staphylococcus aureus* and *Escherichia coli* [120]. Many piezoelectric polymers have also been developed, such as polyvinylidene fluoride (PVDF) and its derivatives: poly-levolactic acid (PLLA), poly-3-hydroxybutyrate-3-hydroxyvalerate (PHBV), etc. They have shown good anti-inflammatory effects or promoted tissue regeneration [121]. A P(VDF-TrFE) piezoelectric film with 2 wt% SrCl_2_ was designed to induce the regeneration of hard dentin tissue. When a piezoelectric film is covered on the pulp or dentin, when the film is deformed by chewing force or other movement, a surface potential is created, inducing stem cell differentiation and release of mineralized matrix. At the same time, the gradual release of Sr can induce dental pulp stem cells to differentiate into odontoblasts [122].

Piezoelectric materials are used to inhibit cariogenic microorganisms, mainly taking advantage of the fact that hard tissues of teeth are frequently stimulated by mechanical forces. Under the stimulation of chewing mechanical force, piezoelectric materials generate microcurrents. Combining piezoelectric materials with restorative materials or appliances is conducive to inhibiting cariogenic microorganisms at relevant sites and accurately antibacterial. Montoya et al. developed a multifunctional dental composite with a BaTiO_3_ piezoelectric nanofiller by taking advantage of the fact that the oral filling body is frequently stimulated by chewing force. The 10% BaTiO_3_-modified dental composite significantly reduced the growth of *Streptococcus mutans* biofilms and promoted ROS generation in cells [93]. Similarly, Shi et al. added nanoparticles containing BaTiO_3_ into invisible appliances and found that their surface antibacterial rate against *Streptococcus mutans* reached 67.39% [94]. In addition, the addition of BaTiO_3_ to denture polymers such as polymethyl methacrylate eradicates fungal biofilms and effectively kills *Candida albicans*, which leads to the production of ROS and the upregulation of the superoxide dismutase gene (*SOD5*) [95]. Polytetrafluoroethylene (PTFE) is a piezoelectric polymer. When external force causes deformation, the charge inside the PTFE will move in the opposite direction to the surface and react with water to generate ROS, which has an effect on bacteria such as *Streptococcus mutans* of up to 80%. It can also degrade organic pigments deposited on the surface of teeth and whiten teeth [96].

Piezoelectric fillers have advantages over traditional antibacterial materials because they provide long-term therapeutic effects without affecting bacterial resistance [123]. Piezoelectric materials also have the advantage of in-depth and precise antibacterial treatment, and are antibacterial materials with great potential. However, there are relatively few studies on the application of piezoelectric materials in caries management. The antibacterial effects of many piezoelectric materials have been confirmed in the medical field. Modifying piezoelectric materials according to the caries microenvironment and then applying them to caries management will be an important research direction.

## 5. Living Materials

The caries microenvironment is a complex system rich in a variety of microorganisms, among which the colonization and aggregation of microorganisms are very complex. The synthetic materials mentioned above have antibacterial effects on cariogenic microorganisms, but they have many shortcomings such as bacterial resistance and insufficient long-term performance. Therefore, living materials modified from microorganisms may be more suitable for the complex microenvironment of caries. The emerging oral health management concept of regulating microbiota through active microorganisms has begun to be applied to the management of oral biofilms with caries [124], including probiotics, bacteriophages, etc. Probiotics and bacteriophages inhibit *Streptococcus mutans* or caries to a certain extent, but they have shortcomings such as poor stability or biosafety. Therefore, researchers have designed engineered living materials that not only respond sensitively to the oral microenvironment but also have stable function [125] (Table 2).

### 5.1. Probiotics

Probiotics are the first traditional living materials to inhibit caries. Most probiotics belong to normal oral flora, and they change microecology by competing with cariogenic bacteria for sites and nutrients [135]. Current research shows that probiotics with anti-caries effects mainly include *Lactobacillus* and *Bifidobacterium* [136]. *Lactobacillus salivarius* can inhibit the formation of cariogenic biofilms of *Candida albicans* and *Streptococcus mutans* and inhibit the morphological transformation of fungi, thereby reducing the pathogenicity of *Candida albicans* and weakening its pathogenic potential [126]. *Lactobacillus rhamnosus* is the most widely used and clinically studied anti-caries probiotic for *Lactobacillus casei*, followed by *Lactobacillus paracasei* [137,138]. Studies have shown that *Lactobacillus rhamnosus* and *Lactobacillus paracasei* inhibit the formation and activity of *Streptococcus mutans* and *Streptococcus oralis* biofilms by producing and delivering H_2_O_2_ [127]. However, *Lactobacillus* is also involved in the occurrence and demineralization of dental caries. This reflects the heterogeneity of probiotics in preventing and treating caries.

*Bifidobacterium Bb12* is also one of the common edible probiotics on the market. Its effect on intestinal microorganisms is relatively clear, but its impact on oral microecology is still controversial. Experiments have shown that eating probiotic yogurt containing *Bifidobacterium Bb12* may have a certain regulatory effect on oral biofilms, and the number of *Streptococcus mutans* and *Lactobacillus* in saliva is significantly reduced [128]. However, research on gut probiotics has shown that probiotics may replace microorganisms that perform important functions, negatively affecting the structure and function of the surrounding microbiota. If the gut barrier is breached, probiotics may enter the systemic circulation, leading to invasive infections [139]. There is also a potential transfer of virulence and antibiotic-resistance genes between different strains through plasmids or transposons, which may lead to harmless probiotics becoming pathogenic and antibiotic-resistant [140]. The safety of long-term application of probiotics is still in doubt and requires caution.

### 5.2. Bacteriophages

Bacteriophages are viruses that infect bacteria, disrupt their metabolism, and ultimately cause host cell lysis [141]. Bacteriophages have the advantages of high specificity, low impact on symbiotic microorganisms, natural non-toxicity, and not producing bacterial drug-resistance mechanisms. Recombinant bacteriophage enzymes have been shown to cause the decomposition of *Streptococcus* [142]. Bacteriophages are divided into lytic and lysogenic. Lytic phages are so named because they lyse host bacteria. Lysogenic phages are transformed into “protophages” by integrating with viral DNA located in the host chromosome. They coexist with the host chromosome for generations, divide, and reproduce with the host chromosome [143].

Bacteriophage APCM01 was obtained from a human sputum sample. After 24 h of contact with *Streptococcus mutans*, a decrease in the metabolic activity of *Streptococcus mutans* biofilms was observed [129]. Lysogenic 4KSM96 extracted from *Streptococcus mutans* significantly inhibited the growth and biofilm formation of *Streptococcus mutans*, and also led to a significant decrease in the proportion of *Streptococcus mutans* in co-culture with other bacterial species. Overall, 4KSM96 showed selective anti-mutant streptococcal activity [130]. Ben-Zaken et al. isolated and characterized a new phage, SMHBZ8, that targets *Streptococcus mutans*. This phage effectively infects and kills plankton and biofilm cultures of *Streptococcus mutans* in vitro, and can also effectively inhibit caries in rats [131,132]. As a highly targeted therapy, bacteriophages inhibit *Streptococcus mutans* very accurately. However, given the complexity of caries biofilms, their disadvantage is that they are too precise and may have weak regulatory effects on cariogenic microbiota. More and more studies have found that bacteria have developed resistance to bacteriophages. Bacteria can form a barrier to bacteriophage adsorption by reducing the availability of phage-bound receptors, and can also block phage adhesion by producing proteins that mask or block phage receptors on the cell surface. Bacteria have also evolved a large number of intracellular proteins that cause abortion in phage infections [144].

### 5.3. Engineered Bacteria

Engineered bacteria are those whose functions are changed in a directional manner due to manual intervention. Recombinant DNA technology can re-edit the genetic material of bacteria to achieve the regulation of the inherent structure and function of bacteria [145]. Engineered bacteria can achieve targeted therapeutic drug delivery, avoid drug degradation during transportation, and deliver therapeutic drugs to parts where bacteria can live, but are difficult to reach through oral or parenteral drug delivery. They can also be programmed to respond to specific stimuli, such as changes in pH or the presence of certain molecules [145]. This programmability allows precise control of drug release. Most of the current research on engineered bacteria is aimed at different diseases, such as diabetes, inflammatory bowel disease, HIV infection, and cancer [146]. Engineered bacteria are selected and designed according to the characteristics of the disease microenvironment to achieve good curative effects.

At present, there are relatively few studies on dental caries with engineered bacteria, mainly focusing on the direction of transgenic *Streptococcus mutans*. Some researchers have genetically edited cariogenic bacteria so that they lose their acid-producing ability while competing with cariogenic bacteria for binding sites and playing an anti-caries role [133]. Transgenic *Streptococcus mutans* can prevent the colonization and growth of *Streptococcus mutans* through alternative therapy. Hillman et al. constructed a BCS3-L1 effector strain based on clinical *Streptococcus mutans* isolates using recombinant DNA technology to delete the gene encoding lactate dehydrogenase in BCS3-L1, making it completely lacking in lactic acid production ability, and designed it to produce a large quantity of a new peptide antibiotic—mutacin1140. This strain expresses genetic stability and good inhibition of the growth of *Streptococcus mutans* [133]. Mao et al. knocked out the *rnc* gene in *Streptococcus mutans* and found that the deletion of this gene disrupted the formation of *Streptococcus mutans* biofilms and reduced cariogenic virulence by inhibiting its downstream VicRKX expression [134]. The combination of this knockout strain and quaternary ammonium has a synergistic effect, inhibits acid production in the *Streptococcus mutans* biofilm, and inhibits secondary caries occurrence [147]. Transgenic *Streptococcus mutans* mainly exerts its anti-caries effect by competing with conventional *Streptococcus mutans* with cariogenic virulence for binding sites. At the same time, because the transgenic *Streptococcus mutans* has no acid production ability, it reduces acid production in the biofilm and inhibits caries. In addition to transgenic *Streptococcus mutans*, designing engineered bacteria based on the characteristics of the caries microenvironment and controlling the precise release of antibacterial drugs is perhaps also an anti-caries strategy in the future. However, the risk of uncontrolled bacterial infection, virulence recovery caused by bacterial gene mutation, interference with the patient microbiome, pharmacodynamic and pharmacokinetic characterization methods, and difficulties in scaling drug production are all difficulties in the future application of engineered bacteria [148].

## 6. Conclusions and Prospects

With the deepening of understanding of cariogenic microbiota, more and more research has abandoned the idea of killing all bacteria and focused on microbial regulation or precise antibacterial function. This review summarizes the characteristics of cariogenic microbiota and traditional and emerging antibacterial materials in recent studies. In addition to the intelligent antibacterial materials such as pH-responsive materials, nanozymes, photoresponsive materials, piezoelectric materials, and living materials mentioned in this article, there are also some emerging materials that focus on the integration of diagnosis and treatment. For example, engineered biomembrane silk fibroin-filled TiO_2_ nanotube array composite membranes (SF–TiO_2_NTbs) are used for *Streptococcus mutans* monitoring, bacterial killing, and biomineralization of dental enamel. They are used to monitor the oral microenvironment, clean *Streptococcus mutans*, and promote long-term remineralization of dental enamel to prevent dental caries [149]. Shi et al. designed a miniaturized, battery-free and wearable dental patch system that electrochemically detects the acidic microenvironment caused by bacterial metabolism and with which fluoride can be delivered locally from electroresponsive drug delivery electrodes for on-demand treatment [150]. The integrated diagnosis and treatment equipment can monitor changes in the caries microenvironment in real time and directly release antibacterial agents to accurately inhibit bacteria. This type of equipment can combat caries through antibacterial activity in the early stage, when the imbalance in caries microecology happens. The new technologies and new materials presented herein provide new strategies for caries prevention and management.

## Figures and Tables

**Table 1:** Effects of intelligent antibacterial materials on cariogenic microbiota.

Types	Agents	Effects on Cariogenic Microbiota
pH-responsive materials	pHly-1NPs [65]	Inhibiting *Streptococcus mutans* and saliva-derived biofilm
	GH12/LH12 [66,67]	Inhibiting *Streptococcus mutans*
	DMAEM/HMAEM [68,69]	Inhibiting *Streptococcus mutans*, *Candida albicans* and saliva-derived biofilm
	DMAEMA-farnesol [70,71]	Inhibiting *Streptococcus mutans*
	DMAEMA-CHX [72]	Inhibiting *Streptococcus mutans*
	PEG-b-PAECOEMA/CA -CHX [73]	Inhibiting *Streptococcus mutans*
	mPEG-b-PDPA-bedaquiline [74]	Inhibiting *Streptococcus mutans*
	PMs@NaF-SAP [75]	Inhibiting *Streptococcus mutans* and saliva-derived biofilm
	PPi-Far-PM [76]	Inhibiting *Streptococcus mutans*
	HTN3-loaded CN [77]	Inhibiting *Streptococcus mutans*
	CHX-loaded/MSN-PGA [78]	Inhibiting *Streptococcus mutans* and *Staphylococcus aureus*
	Ag-MSNs@CHX [79]	Inhibiting *Streptococcus mutans*
Nanozymes	CAT-NP [80]	Inhibiting *Streptococcus mutans*, degrading biofilm matrix
	Dex-IONP-GOx [81]	Inhibiting *Streptococcus mutans*, no influence on *Streptococcus oralis*
	Dex-NZM [82]	Inhibiting *Streptococcus mutans*, degrading biofilm matrix
	SnF_2_-Fer [83]	Inhibiting *Streptococcus mutans*
	DNA-encoded IONPs [84]	Identifying and inhibiting *Streptococcus mutans* and *Lactobacillus acidophilus*
	GLA/GS [85]	Inhibiting *Streptococcus mutans*
Photoresponsive materials	FePAgPG [86]	Inhibiting *Streptococcus mutans*
	BP@CP5 [87]	Inhibiting *Streptococcus mutans* and *Streptococcus sanguis*
	MPP-Ce6 [88]	Inhibiting *Streptococcus mutans*, *Streptococcus sobrinus* and *Streptococcus sanguis*
	MB-CNP/MB-TiO_2_NP [89]	Inhibiting *Streptococcus mutans*
	CS/SA NPs-CUR [90]	Inhibiting *Streptococcus mutans*, no influence on *Candida albicans*
	PS-NP@CIP [91]	Inhibiting *Streptococcus mutans*
	GFZ [92]	Inhibiting *Streptococcus mutans*
Piezoelectric materials	BaTiO_3_ [93,94,95]	Inhibiting *Streptococcus mutans* and *Candida albicans*
	PTFE [96]	Inhibiting *Streptococcus mutans*

**Table 2:** Effects of living materials on cariogenic microbiota.

Types	Agents	Effects on Cariogenic Microbiota	Advantages	Disadvantages
Probiotics	*Lactobacillus salivarius* [126]	Inhibiting *Streptococcus mutans* and *Candida albicans*	Effectively inhibiting cariogenic biofilm	Probably transferring to pathogenic and antibiotic-resistant strains and affecting biological safety
	*Lactobacillus rhamnosus* and *Lactobacillus paracasei* [127]	Inhibiting *Streptococcus mutans* and *Streptococcus oralis*		
	*Bifidobacterium Bb12* [128]	Inhibiting *Streptococcus mutans* and *Lactobacillus*		
Bacteriophages	APCM01 [129]	Inhibiting *Streptococcus mutans*	Accurately inhibiting *Streptococcus mutans*	Developing bacteria with resistance to bacteriophages
	4KSM96 [130]	Inhibiting *Streptococcus mutans*		
	SMHBZ8 [131,132]	Inhibiting *Streptococcus mutans*		
Engineered bacteria	BCS3-L1 [133]	Inhibiting *Streptococcus mutans*	Effectively inhibiting *Streptococcus mutans*	Inexact biosecurity caused by uncontrolled bacterial infection or virulence recovery
	rnc-deficient strain of *Streptococcus mutans* [134]	Inhibiting *Streptococcus mutans*		

## Data Availability

No new data were created or analyzed in this study. Data sharing is not applicable to this article.
